# Sex‐Differential Trajectories of Domain‐Specific Associations Between Autistic Traits and Co‐Occurring Emotional‐Behavioral Concerns in Autistic Children

**DOI:** 10.1002/aur.70018

**Published:** 2025-03-14

**Authors:** Yun‐Ju Chen, Thomas W. Frazier, Peter Szatmari, Eric Duku, Annie E. Richard, Isabel M. Smith, Lonnie Zwaigenbaum, Rachael Bedford, Connor M. Kerns, Anat Zaidman‐Zait, Terry Bennett, Mayada Elsabbagh, Tracy Vaillancourt, Stelios Georgiades

**Affiliations:** ^1^ Offord Centre for Child Studies McMaster University Hamilton Ontario Canada; ^2^ Department of Occupational Therapy and Graduate Institute of Behavioral Sciences Chang Gung University Taoyuan Taiwan; ^3^ Department of Psychiatry Chang Gung Memorial Hospital at Linkou Taoyuan Taiwan; ^4^ Department of Psychology John Carroll University University Heights Ohio USA; ^5^ Centre for Addiction and Mental Health Toronto Ontario Canada; ^6^ The Hospital for Sick Children Toronto Ontario Canada; ^7^ University of Toronto Toronto Ontario Canada; ^8^ Autism Research Centre, IWK Health Centre Halifax Nova Scotia Canada; ^9^ Department of Pediatrics Dalhousie University Halifax Nova Scotia Canada; ^10^ Department of Pediatrics University of Alberta Edmonton Alberta Canada; ^11^ Department of Psychology University of Bath Bath UK; ^12^ Department of Psychology University of British Columbia Vancouver British Columbia Canada; ^13^ Constantine School of Education Tel Aviv University Tel Aviv Israel; ^14^ Department of Neurology and Neurosurgery McGill University Montreal Quebec Canada; ^15^ Department of Education and Psychology University of Ottawa Ottawa Ontario Canada

**Keywords:** autism, co‐occurring emotional/behavioral concerns, longitudinal, measurement invariance, time‐varying associations

## Abstract

Assessing autistic traits alongside co‐occurring emotional/behavioral concerns (EBCs) is challenging due to their overlap in clinical presentations, which can vary by age and sex. This study aimed to investigate domain‐specific associations between autistic traits and EBCs–including anxiety, affective, attention‐deficit/hyperactivity, and oppositional‐defiant problems–across childhood in autistic boys and girls. We prospectively followed 389 children (84% male) diagnosed with autism at ages 2–5 years, using the Social Responsiveness Scale (SRS) and Child Behavior Checklist (CBCL) across eight timepoints until age 12. Moderated nonlinear factor analysis was used to identify and adjust for measurement non‐invariance of SRS items by age, sex, and EBCs. The adjusted scores were then used for sex‐moderated time‐varying modeling of associations between autistic traits and EBCs. Several SRS items in the domains of social‐interaction difficulties and repetitive mannerisms showed significant intercept bias by age and level of co‐occurring anxiety and ADHD (effect size *r* > 0.20). In autistic boys, strong associations were observed between social‐communication difficulties and EBCs around ages 7–9, which tended to diminish in late childhood. In contrast, autistic girls showed stable or intensifying associations, particularly with anxiety, into late childhood. Results revealed significant associations between autistic traits and EBCs after addressing item‐level measurement biases. The varying associations over time highlight the importance of continuous monitoring to promptly address autistic children's sex‐differential mental health needs. These findings emphasize the benefits of refining behavioral constructs and adopting a nuanced developmental approach to identify critical periods of symptom coupling/decoupling for informing evaluation and service provision.

**Summary**

- In this study, we tracked autistic children from ages 2 to 12 using caregiver questionnaires to understand how autism traits relate to anxiety, mood problems, inattention/hyperactivity, and oppositional behavior over time.
- We found that caregivers' ratings of autism traits could be influenced by the child's age and the presence of anxiety and inattention/hyperactivity symptoms, introducing bias that may complicate the interpretation of the scores.
- After addressing biases related to child characteristics, we observed that social‐communication difficulties were strongly associated with co‐occurring problems, particularly mood problems and ADHD, around ages 7–9 in autistic boys, but this association diminished afterward.
- In contrast, these associations tended to increase beyond age 9 for autistic girls, especially with anxiety symptoms.
- These findings highlight the importance of continuous monitoring and individualized support strategies to address the different mental health needs of autistic boys and girls across stages of life.

## 1 Introduction

Co‐occurring mental health and neurodevelopmental conditions, such as anxiety, depression, and attention‐deficit/hyperactivity disorder (ADHD), are prevalent in autistic people, profoundly impacting various aspects of their daily lives (Lai 2023; Rosen et al. 2018). Differentiating between autism symptoms and emotional/behavioral concerns (EBCs) is central to making an appropriate diagnosis, which in turn is important for the identification of appropriate services and supports, assisting in personalized healthcare (Lord et al. 2022; Waizbard‐Bartov et al. 2023). However, capturing autistic traits alongside EBCs is challenging due to their overlap in etiology and clinical presentations (Hawks and Constantino 2020; Hoekstra et al. 2007; Rosen et al. 2018), as evidenced by the well‐documented associations between levels of autistic traits and EBCs observed in both clinical and nonclinical samples across the lifespan (Hallett et al. 2009; Hus et al. 2013; Lundström et al. 2011; Polderman et al. 2014). Such symptom covariation or “coupling” often complicates the process of differential diagnosis (e.g., diagnostic overshadowing), resulting in delays in timely diagnoses crucial for accessing necessary services (Mahony et al. 2023; McCauley et al. 2020). Despite these challenges, understanding how core autistic traits are differentially associated with more general measures of EBCs can have important implications for addressing related emotional and behavioral challenges that often substantially impact autistic people's daily functioning and quality of life (Lord et al. 2022; Menezes and Mazurek 2021).

The association between autism and co‐occurring EBCs can be further complicated by their intricate interactions with individual factors such as age and sex/gender (McCauley et al. 2020; Supekar et al. 2017). For instance, cross‐sectional evidence has shown generally stronger associations between autistic and ADHD traits in school‐aged children than in preschoolers (Visser et al. 2016), and weaker links in adults than in children (Lundström et al. 2011). Longitudinal evidence indicated that these associations peak during adolescence (Hartman et al. 2016), suggesting a potential quadratic trend that might not be well captured by cross‐sectional data. Regarding internalizing conditions, a previous cross‐sectional study reported weaker links between autistic traits and anxiety symptoms in autistic adolescents compared to younger children (Vasa et al. 2013). This age‐related pattern aligns with longitudinal evidence indicating stable concurrent associations between anxiety and autistic traits (particularly insistence on sameness) throughout early childhood, followed by their decreased correlation in late childhood (Baribeau et al. 2023). While both cross‐sectional and longitudinal data can be utilized to address age effects on the associations, cross‐sectional designs do not inform about within‐person changes over time and may yield different findings than longitudinal studies (Chen et al. 2023; Georgiades et al. 2017; Sliwinski et al. 2010). Moreover, the existing longitudinal evidence is restricted to the discrete age points at which data were collected, possibly obscuring more nuanced and continuous patterns in the association.

Regarding the moderating effect of sex assigned at birth (hereafter, “sex”), differential prevalence of co‐occurring conditions in autistic males and females has been widely reported in the literature, with autistic males more prone to diagnoses of externalizing conditions and autistic females more likely to have internalizing conditions (Angell et al. 2021; Gu et al. 2023; Wodka et al. 2022). Further, prior research generally suggests stronger associations between autistic traits and internalizing problems in autistic females than in males (Barnett et al. 2021; Kreiser and White 2015; Zhao et al. 2020), while the links were stronger for externalizing and attention problems in autistic males compared to females (Hollingdale et al. 2023; Hsiao et al. 2013). A recent SPARK cohort study found stronger associations between co‐occurring anxiety or ADHD diagnosis and social‐communication difficulties in autistic females under 12 years old, compared to males and older females, whereas in adolescent females, the associations were stronger with repetitive and restrictive behaviors (RRBs) (Wodka et al. 2022). These findings thus highlight the need for systematic investigations into the varying relationships between autistic traits and EBCs, considering both age and sex. Particularly, their potential moderating effects on finer‐grained domains (e.g., social‐communication and RRB instead of overall autistic traits) using longitudinal data remain understudied. Such evidence can shed light on the specific domains and subpopulations exhibiting more notable symptom “coupling” as well as the critical developmental period that necessitates additional targeted supports in addressing the emotional and behavioral challenges linked to autism.

**FIGURE 1:**
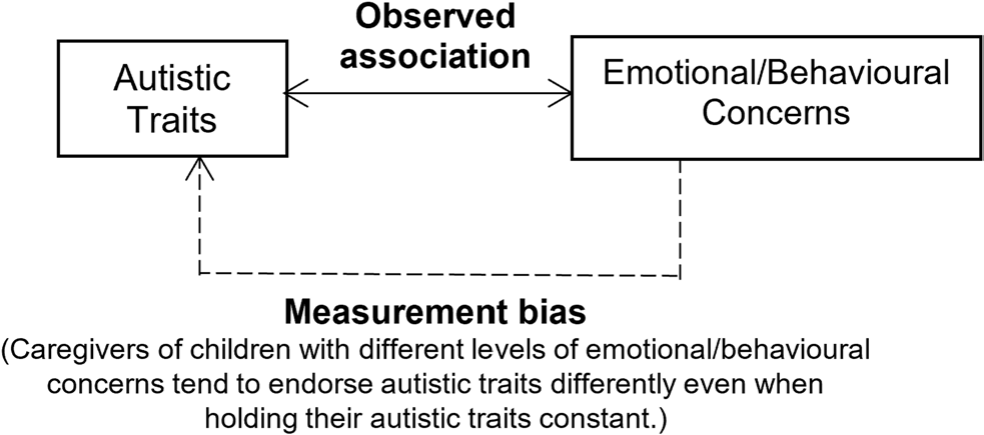
Distinguishing measurement biases related to co‐occurring emotional/behavioral concerns from observed associations.

Another layer of complexity pertains to symptom measurement in autism, which often relies on proxy‐report or self‐report questionnaires. The default assumption when using observed scores for statistical inference is that questionnaires accurately capture the behavioral traits they were designed to measure. However, this assumption often proves insufficient, as individual factors such as demographics (e.g., age and sex) and clinical characteristics (e.g., co‐occurring symptoms) can influence respondents' tendencies to endorse certain responses when assessing the behavioral traits of interest (e.g., autistic traits in the current study), leading to measurement non‐invariance or bias. When such issues arise at the item level of a measurement, they are referred to as differential item functioning (DIF). A previous study examining DIF in a parent‐report measure of anxiety found varying thresholds for certain social anxiety items based on different levels of autistic traits when applied to autistic youth (Schiltz and Magnus 2021). This indicates potential measurement‐related confounding between autistic traits and co‐occurring EBCs, warranting further investigations in the current study. To address this, moderated nonlinear factor analysis (MNLFA) offers a flexible approach to account for multiple sources of measurement bias by testing and adjusting for DIF across continuous covariates (e.g., age and symptom levels), which is a feature superior to the conventional multi‐group confirmatory factor analysis (Bauer 2017). In the current study, we aimed for an in‐depth longitudinal exploration of measurement bias to evaluate the associations between autism traits and EBCs (see Figure 1).

Overall, current evidence regarding the associations between autism traits and EBCs reveals several gaps, including (1) age‐varying associations based on longitudinal data, (2) identification and adjustment of item‐level measurement biases, and (3) sex‐differential associations across domains. As such, in the current study, we aimed to investigate and address these empirical gaps systematically through the following research aims:

- Aim 1: To evaluate and adjust for the impact of measurement biases related to child's age, sex, and co‐occurring EBCs (anxiety, affective, ADHD, and oppositional defiant problems as measured by the Child Behavior Checklist [CBCL] DSM‐oriented scales) on parental reports of autistic traits in autistic children measured by the Social Responsiveness Scale (SRS),
- Aim 2: To estimate the age‐varying associations between domains of autistic traits (i.e., SRS latent construct scores adjusted for DIF) and EBCs in autistic children, and to identify age periods that exhibit significant sex differences.

The first research aim is exploratory due to the absence of prior evidence of measurement bias by EBCs in assessing autistic traits. Regarding the second aim, despite a lack of evidence on this inquiry, we expect that the associations between autistic traits and EBCs will fluctuate across age and vary by behavioral domain. Particularly, entering school age may be a crucial period for the strengthening of these links, given the increased rates of diagnosed mental health conditions (Salazar et al. 2015; Soke et al. 2018) and the heightened environmental demands associated with more emotional‐behavioral and functional challenges among autistic children (Chen et al. 2023; Lai et al. 2020). Concerning the moderating role of sex, we expect the overall associations between autistic traits and externalizing problems to be stronger in boys, while the associations with internalizing conditions would be stronger in girls, though the patterns may vary over time. Given prior reports of increasingly apparent social difficulties and vulnerability to co‐occurring conditions in autistic females during late childhood and adolescence (Jamison et al. 2017; Lai et al. 2022), we posit that the strength of associations will be greater in autistic girls than in boys during late childhood, with potential variations across behavioral domains.

## 2 Method

### 2.1 Participants

The participants in the current study were drawn from an inception cohort diagnosed with autism at ages 2 to 5 years through the *Pathways in ASD* study, a Canadian longitudinal cohort study across five sites: Halifax, Montreal, Hamilton, Edmonton, and Vancouver (see Szatmari et al. 2015 for inclusion and exclusion criteria). Following enrollment within 4 months of diagnosis, children underwent repeated behavioral assessments at 6 and 12 months post‐enrollment, with subsequent data collection occurring around ages 6, 8, 9, 10, and 11 years. The current sample included 389 children from the full *Pathways in ASD study* cohort (*N* = 421) who had at least one SRS‐CBCL data point across eight data waves, yielding a total of 1944 observations (see Table 1 for sample characteristics). The descriptive statistics for primary measures by time point are shown in Table S1.

**TABLE 1:** Sample characteristics at baseline.

	≥ 1 wave (*N* = 389)	≥ 4 waves (*N* = 277)	< 4 (*n* = 112) versus ≥ 4 waves (*n* = 277)
Age in years at diagnosis and assessments, mean (SD)			T‐test Statistics (*p*)
Diagnosis	38.3 (8.8)	38.7 (8.7)	1.50 (0.13)
SRS	41.0 (9.3)	41.3 (9.2)	0.97 (0.33)
CBCL	41.0 (9.3)	41.2 (9.1)	0.86 (0.39)
Nonverbal IQ at diagnosis^a^	56.9 (26.9)	58.1 (27.4)	1.26 (0.21)
Demographic characteristics			Odds ratio (*p*)
Sex assigned at birth, *N* (% male)	327 (84.1)	239 (86.3)	0.58 (0.06)
Site, *N* (%)			
Halifax	53 (13.6)	35 (12.6)	1.32 (0.37)
Montreal	129 (33.2)	101 (36.5)	0.58 (0.03)*
Hamilton	59 (15.2)	34 (12.3)	2.05 (0.01)*
Vancouver	90 (23.1)	69 (24.9)	0.70 (0.19)
Edmonton	58 (14.9)	38 (13.7)	1.37 (0.30)
Household income, *N* (%)			
Less than $30,000	62 (15.9)	41 (14.8)	1.55 (0.14)
$30,000–$80,000	163 (41.9)	119 (43.0)	1.06 (0.81)
$80,000 or more	139 (35.7)	108 (40.0)	0.71 (0.17)
Primary caregiver's education, *N* (%)			
High school or less	50 (13.5)	31 (11.4)	1.87 (0.05)
Some post‐secondary education	163 (44.1)	121 (44.5)	0.94 (0.78)
Bachelor's degree or higher	157 (42.4)	120 (44.1)	0.77 (0.28)
Primary caregiver's ethnicity, *N* (%)			
White	276 (74.2)	204 (74.2)	1.00 (0.99)
South/Southeast Asian	27 (7.2)	20 (7.3)	0.99 (0.98)
East Asian	20 (5.4)	18 (6.5)	0.30 (0.11)
Arab	13 (3.5)	9 (3.3)	0.27 (0.70)
Black	13 (3.5)	9 (3.3)	0.27 (0.70)
Indigenous	9 (2.4)	4 (1.4)	3.68 (0.06)
Latinx	7 (1.9)	6 (2.2)	0.47 (0.48)
Other	7 (1.9)	5 (1.8)	1.14 (0.88)

*Note*: *p < 0.05, **p < 0.01.

^a^ Nonverbal IQ was measured by the Merrill‐Palmer‐Revised Scales cognitive subscale (standard score mean = 100, SD = 15, range = 10–160).

### 2.2 Measures

#### 2.2.1 Social Responsiveness Scale (SRS; Constantino and Gruber 2005)

The SRS is a 65‐item parent‐report measure of autistic traits with well‐established psychometric properties in both clinical and general populations. Each item is rated on the Likert scale from 1 (not true) to 4 (almost always true) reflecting the frequency of the observed behavior. The positively worded items are reverse‐coded for scoring so that higher scores indicate a higher level of autistic traits. Although the total score, assuming a single‐factor structure (Constantino et al. 2004), has been commonly utilized in clinical settings, prior research on the construct validity of the SRS generally revealed two to five latent factors capturing social communication and restricted/repetitive behaviors in autistic and non‐autistic samples across age spans (e.g., Chan et al. 2017; Frazier et al. 2014; Uljarević et al. 2020). Given the primary inquiries on domain‐level associations, we utilized item‐level SRS data to establish constructs empirically for the current longitudinal autistic sample (see the Section 2.3.1).

#### 2.2.2 Child Behavior Checklist for Ages 1.5–5 and 6–18 (CBCL/1.5–5 and 6–18; Achenbach and Edelbrock 1991)

The CBCL is a proxy‐report measure of children's behavioral and emotional problems, offering multiple norm‐referenced subscales within the problem behavior scale. These include DSM‐oriented scales specifically designed to aid in the differential diagnostic process. For this study, we focused on the four DSM‐oriented scales shared across the 1.5–5 and 6–18 age versions: anxiety, affective, attention deficit/hyperactivity (ADHD), and oppositional‐defiant (ODD) problems. Previous research has supported the test–retest and inter‐rater reliability, as well as the discriminative, convergent, and predictive validity of the DSM‐oriented scales in large clinical samples (Bellina et al. 2013; Nakamura et al. 2009). We used T‐scores (standardized with a mean of 50 and a standard deviation of 10) from these subscales to ensure comparability of scores across age.

### 2.3 Statistical Analyses

#### 2.3.1 Construct Validity of the SRS: Confirmatory Factor Analysis (CFA)

To validate the SRS constructs to be used in our primary analyses, a series of CFA models with different factor structures was fitted separately to three age samples: ages 2–5 (*n* = 388), ages 6–8 (*n* = 279), and ages 9–12 (*n* = 249). For each age sample, each participant contributed only one randomly selected data‐wave point. The specification of the factor structures was based on the SRS manual (Constantino and Gruber 2012), prior literature (Frazier et al. 2014), and the evaluation of modification indices and clinical interpretations (see Figure S1 for the item‐factor correspondence across models of various factor structures), including the following five models:

- One‐factor model: all items loaded onto one factor
- Two‐factor model: DSM‐5 Social‐Communication and RRB scales are described in the SRS manual (Constantino and Gruber 2012)
- Five‐factor model A: treatment scales described in the SRS manual (Constantino and Gruber 2012)
- Five‐factor model B: factor structure proposed by Frazier et al. (2014)
- Five‐factor model C: factor structure modified from Frazier et al. (2014)

Each item was exclusively loaded to one latent factor without any cross‐loading permitted. The factor analyses were conducted with robust maximum likelihood estimation in Mplus 8.4 (Muthén and Muthén 2018).

#### 2.3.2 Measurement Invariance Testing of the SRS: Moderated Nonlinear Factor Analysis (MNLFA)

**FIGURE 2:**
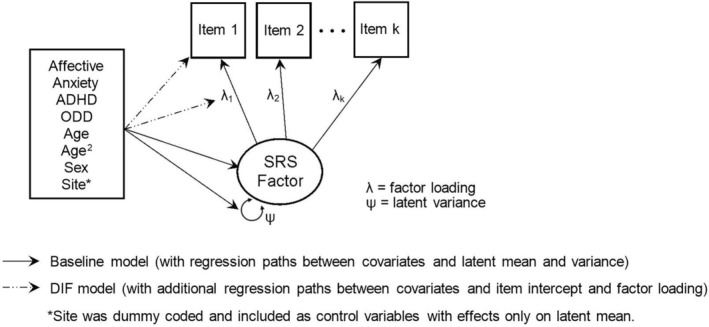
MNLFA model specification.

Given the longitudinal nature of the current sample, MNLFA model parameters were estimated on three calibration samples pseudo‐randomly drawn from independent observations (each *n* = 393 with identical age distribution to the full longitudinal sample) item by item, following the steps described in the previous studies examining DIF using longitudinal data (Gottfredson et al. 2019; Sifre et al. 2021). In the initial phase (the baseline model as shown in Figure 2), we assessed the covariate effects of continuous age, sex, co‐occurring EBCs (T‐scores from the four CBCL subscales), and the quadratic age term (age^2^) on the latent mean and variance of each SRS factor. Study site was dummy‐coded and included as a control variable only with effects on the latent mean. We retained model parameters with a significance level below *p* < 0.10, while trimming those exceeding this threshold. Next, the covariate effects were introduced to the item intercept and factor loading for the identification of DIF (the DIF model in Figure 2). Model parameters with *p* < 0.05 identified across the calibration samples were retained in the final model, which was used for factor score estimation in the full longitudinal sample (*n* = 1944). A full‐information maximum likelihood (FIML) estimator was applied throughout this part of the analysis to accommodate missing data. The derived factor scores that accounted for DIF (DIF‐adjusted SRS factor scores) were then used in the subsequent time‐varying effect modeling.

#### 2.3.3 Trajectories of SRS‐CBCL Associations: Time‐Varying Effect Modeling (TVEM)

The second research aim about age‐varying associations was addressed using TVEM, which allows for modeling *change in association* (in the form of regression coefficients or slopes) as a flexible, nonparametric function of time (Lanza and Linden‐Carmichael 2021; Tan et al. 2012). Autistic traits (i.e., DIF‐adjusted SRS factor scores) were regressed simultaneously on EBCs (i.e., CBCL subscale T‐scores), sex, and their interaction term, with the coefficients varying as a function of age. The age‐varying associations were evaluated for each pair of the SRS construct and CBCL subscale, yielding a total of 20 TVEM models. Listwise deletion was applied at the observation level (e.g., a participant providing data on 6 out of possible 10 repeated observations would contribute 6 observations to the estimation), with all available SRS‐CBCL paired data retained in the analysis. The degree of smoothness (or number of knots) for each coefficient function was determined based on comparisons of pseudolikelihood Bayesian information criterion (BIC) values from models with zero through five knots with unpenalized B‐spline estimation (Tan et al. 2012). Statistically significant effects are indicated when the 95% confidence interval around a time‐specific regression coefficient excludes zero. All TVEM models were fitted using the R package “tvem” (Dziak et al. 2021).

## 3 Results

### 3.1 Construct Validity of the SRS

The results of the CFA models fitted to the five proposed models across independent age samples are presented in Table 2. All models generally showed a better fit to the older age groups (CFI/TLI = 0.88–0.94, RMSEA = 0.03–0.05) than to the age 2–5 group (CFI/TLI = 0.72–0.90, RMSEA = 0.04–0.06), indicating that multiple‐factor solutions for autistic traits were favored in the younger age range. The best fitting model was the Five‐Factor Model C (CFI/TLI = 0.90–0.94, RMSEA = 0.04), with model fit superior to that of the other four models (CFI/TLI = 0.72–0.93, RMSEA = 0.04–0.06). The five factors consist of *Social Avoidance* (F1; 9 items), *Social–Emotional (Un)Awareness* (F2; 15 items), *Social‐Interaction Difficulties* (F3; 12 items), *Insistence on Sameness* (F4; 15 items), and *Repetitive Mannerisms* (F5; 12 items). The factor loadings (average values across items = 0.57–0.70) and composite reliability (*ω* = 0.86–0.92) of the Five‐Factor Model C are presented in Table S2. The inter‐factor correlations range from *r = 0*.29 to 0.73 (see Table S3).

**TABLE 2:** Model fit statistics of confirmatory factor analyses for the SRS.

	1‐factor model			2‐factor model (DSM‐5)			5‐factor model A (treatment)			5‐factor model B (Frazier et al. 2014)			5‐factor model C (modified from Frazier et al. 2014)^a^		
Sample	1	2	3	1	2	3	1	2	3	1	2	3	1	2	3
CFI	0.731	0.886	0.912	0.750	0.897	0.919	0.736	0.891	0.916	0.788	0.912	0.934	0.900	0.938	0.940
TLI	0.722	0.882	0.909	0.742	0.893	0.916	0.726	0.887	0.913	0.780	0.909	0.931	0.896	0.936	0.938
RMSEA [90% CI]	0.064 [0.061, 0.066]	0.055 [0.052, 0.058]	0.050 [0.047, 0.054]	0.061 [0.059, 0.063]	0.052 [0.049, 0.055]	0.048 [0.045, 0.052]	0.063 [0.061, 0.065]	0.054 [0.051, 0.057]	0.049 [0.046, 0.053]	0.056 [0.054, 0.059]	0.048 [0.045, 0.051]	0.044 [0.040, 0.047]	0.039 [0.036, 0.041]	0.041 [0.037, 0.044]	0.042 [0.038, 0.045]
*χ* ^2^	4850.5	3489.8	3089.2	4636.3	3333.2	2995.0	4781.6	3402.2	3017.1	4208.5	3112.8	2784.0	2976.9	2743.6	2697.5
df	1890	1890	1890	1889	1889	1889	1880	1880	1880	1880	1880	1880	1880	1880	1880

*Note*: sample 1: Independent sample drawn from waves 1–3 (*n* = 388, ages 2–5); sample 2: independent sample drawn from waves 4–5 (*n* = 279, ages 6–8); sample 3: independent sample drawn from waves 6–8 (*n* = 249, ages 9–12).

^a^ Two items were dropped: Item 43 (separates easily from caregivers) was dropped due to its weak factor loading across models. Item 11 (“has good self‐confidence”) was dropped due to its high residual covariance with item 3 (“seems self‐confident when interacting with others”).

### 3.2 Aim 1: Measurement Invariance/Differential Item Functioning of the SRS by Age, Sex, and Co‐Occurring EBCs

The item‐level MNLFA results for each SRS factor are presented in the Supporting Information: Spreadsheet 1. To summarize the results, we converted the beta coefficient estimates to Pearson's *r* for each item, quantifying the degree the item intercept and loading differ across age, sex, and levels of co‐occurring EBCs (see Olino et al. 2021 for a similar approach). Pearson's *r* correlation coefficients were then averaged across the calibration samples and items for each of the SRS factors (see Table 3 for construct‐level and Table S4 for item‐level summarized results). The average magnitude of correlations with age across items was relatively large for intercepts (*r* = 0.071–0.140) compared to factor loadings (*r* = 0.038–0.048). The intercept bias by age was larger for F3 (*Social‐Interaction Difficulties*) and F5 (*Repetitive Mannerisms*). Five items showed relatively large intercept bias by age (*r* > 0.200 which indicates practical significance; Ferguson 2009): item 29 (*is regarded by other children as odd or weird* [F5]; *r* = 0.299), item 57 (*other children do not like to play with him/her*; *r* = 0.292), item 47 (*is too silly or laughs inappropriately* [F5]; *r* = 0.269), item 9 (*clings to adults, seems too dependent on them*; *r* = 0.212), and item 41 (*wanders aimlessly from one activity to another* [F5]; *r* = 0.202). These indicate that caregivers of older children were more inclined to endorse items 29, 47, and 57, whereas items 9 and 41 were more likely to be endorsed by caregivers of younger children when controlling for autistic traits. The associations with quadratic age and sex were overall small for both intercepts (*r* = 0.039–0.072) and factor loadings (*r* = 0.027–0.049).

**TABLE 3:** Magnitude of associations (Pearson's *r*) between covariates (age, sex, and level of co‐occurring emotional/behavioral concerns) and structural parameters for autistic traits for each SRS latent factor.

Covariates	SRS latent factors	Intercept bias mean (SD)	Max. intercept bias	Loading bias mean (SD)	Max. loading bias
Age	F1	0.075 (0.039)	0.154	0.039 (0.017)	0.070
	F2	0.071 (0.037)	0.137	0.039 (0.015)	0.079
	**F3**	**0.140 (0.070)**	**0.292**	0.038 (0.023)	0.097
	F4	0.080 (0.048)	0.167	0.048 (0.025)	0.100
	**F5**	**0.112 (0.099)**	**0.299**	0.047 (0.019)	0.085
Age^2^	F1	0.039 (0.009)	0.059	0.044 (0.016)	0.068
	F2	0.063 (0.029)	0.129	0.042 (0.014)	0.063
	F3	0.072 (0.033)	0.113	0.041 (0.020)	0.076
	F4	0.050 (0.021)	0.092	0.049 (0.024)	0.088
	F5	0.052 (0.026)	0.102	0.040 (0.016)	0.079
Male	F1	0.046 (0.015)	0.069	0.044 (0.021)	0.074
	F2	0.045 (0.036)	0.167	0.033 (0.018)	0.073
	F3	0.040 (0.018)	0.076	0.027 (0.014)	0.057
	F4	0.040 (0.021)	0.085	0.039 (0.023)	0.092
	F5	0.051 (0.034)	0.134	0.045 (0.023)	0.074
Affective	F1	0.055 (0.022)	0.105	0.040 (0.019)	0.088
	F2	0.046 (0.026)	0.087	0.037 (0.017)	0.075
	F3	0.045 (0.030)	0.099	0.044 (0.020)	0.078
	F4	0.055 (0.031)	0.113	0.036 (0.021)	0.098
	F5	0.050 (0.022)	0.101	0.045 (0.021)	0.088
Anxiety	F1	0.047 (0.030)	0.103	0.037 (0.013)	0.064
	F2	0.055 (0.042)	0.173	0.049 (0.022)	0.090
	**F3**	**0.076 (0.071)**	**0.282**	0.050 (0.031)	0.127
	F4	0.042 (0.027)	0.096	0.040 (0.015)	0.079
	F5	0.039 (0.021)	0.077	0.040 (0.020)	0.068
ADHD	F1	0.047 (0.021)	0.082	0.045 (0.021)	0.071
	F2	0.064 (0.031)	0.150	0.037 (0.014)	0.058
	F3	0.056 (0.021)	0.091	0.049 (0.028)	0.117
	F4	0.048 (0.031)	0.144	0.043 (0.016)	0.074
	**F5**	**0.081 (0.062)**	**0.249**	0.038 (0.017)	0.067
ODD	F1	0.047 (0.025)	0.087	0.034 (0.014)	0.069
	F2	0.054 (0.023)	0.087	0.031 (0.021)	0.075
	F3	0.050 (0.033)	0.130	0.044 (0.024)	0.087
	F4	0.051 (0.031)	0.138	0.034 (0.015)	0.054
	F5	0.035 (0.018)	0.065	0.050 (0.015)	0.074

*Note*: The effect sizes shown in this table were averaged across items and three calibration samples. Please see Table S4 for item‐level results. Rows with a maximum magnitude of item‐level bias *r* > 0.20 are bolded. F1 = social avoidance, F2 = social–emotional unawareness, F3 = social‐interaction difficulties, F4 = insistence on sameness. F5 = repetitive mannerisms (F5; 12 items).

As for co‐occurring EBCs, the magnitude of associations was also small for intercepts (*r* = 0.035–0.081) and loadings (*r* = 0.031–0.050), with stronger average associations observed between the intercept of F3 (*Social‐Interaction Difficulties*) and anxiety symptoms and between the intercept of F5 (*Repetitive Mannerisms*) and ADHD symptoms. Specifically, there were two items with relatively large intercept bias by EBCs (*r* > 0.200): item 9 (*clings to adults, seems too dependent on them*) was associated with anxiety symptoms (*r* = 0.282), and item 41 (*wanders aimlessly from one activity to another* [F5]) was associated with ADHD symptoms (*r* = 0.249). Both items were more likely to be endorsed by caregivers of children with higher levels of anxiety or ADHD symptoms when controlling for autistic traits.

### 3.3 Aim 2: Trajectories of Domain‐Level Associations Between Autistic Traits and EBCs

Age‐varying associations without sex stratification are shown in Figure S2. Overall, autistic traits were positively correlated with EBCs over time across all domains. The associations between SRS social domains (i.e., *Social Avoidance*, *Social–Emotional Unawareness*, and *Social‐Interaction Difficulties*) and CBCL domains showed quadratic trends, with inflection points observed at certain ages. Notably, a substantial decrease was seen in the association between social‐interaction difficulties and affective problems from age 2 (*β* = 0.072 [95% CI = 0.047–0.097]) to age 12 (*β* = 0.032 [95% CI = −0.004–0.069]), with a concave downward trend peaking around age 8 (*β* = 0.082 [95% CI = 0.071–0.093]). Another significant decrease was observed in the association between social–emotional unawareness and ADHD symptoms from age 2 (*β* = 0.070 [95% CI = 0.037–0.103]) to age 12 (*β* = 0.011 [95% CI = −0.024–0.045]). The only notable increase was found in the association between social–emotional unawareness and ODD from age 2 (*β* = 0.014 [95% CI = −0.015–0.043]) to age 12 (*β* = 0.046 [95% CI = 0.006–0.087]). Compared to social‐communication domains (*β* = 0.013–0.066), RRB domains exhibited generally stronger associations (*β* = 0.055–0.092) with all CBCL domains, particularly during the preschool age range. A large decrease was observed in the association between repetitive mannerisms and affective problems from age 2 (*β* = 0.105 [95% CI = 0.078–0.132]) to age 12 (*β* = 0.011 [95% CI = −0.035–0.056]), as well as between repetitive mannerisms and ADHD symptoms from age 2 (*β* = 0.114 [95% CI = 0.074–0.154]) to age 12 (*β* = 0.039 [95% CI = −0.003–0.081]). The TVEM results are summarized in Table S5.

**FIGURE 3:**
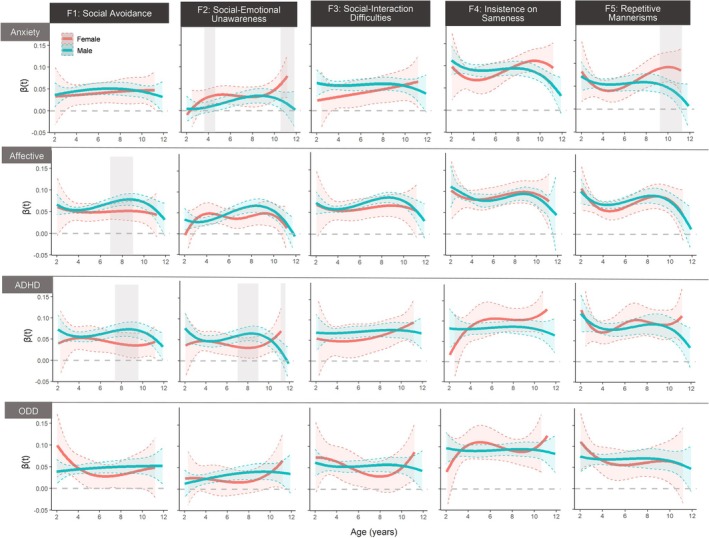
Age‐varying associations between CBCL domains (row) on SRS factors (column) by sex. *Note*: The y‐axis represents the age‐varying main effects of each CBCL subscale on each SRS factor, which were modeled as spline regression coefficients and confidence intervals, with all available data from each individual at each age point used for parameter estimation. Significant effects are indicated when the 95% confidence intervals do not overlap with zero. Shaded areas in light gray represent significant sex interaction effects within specified age ranges. The TVEM estimates used to generate these figures are available in Supporting Information: Spreadsheet 2, which includes indications of significance at *α* = 0.0025, adjusted using the Bonferroni correction for multiple comparisons.

Examining the age‐varying associations stratified by sex, the results revealed several significant moderation effects of sex for specific SRS‐CBCL associations at certain ages. As highlighted by the shaded areas in Figure 3, the association between social avoidance and affective problems peaked between ages 7 and 9 in boys, but not in girls (average interaction effect: *β* = 0.025 [95% CI = 0.001–0.048]). A similar pattern was found in the association between social avoidance and ADHD symptoms, with the peak exclusively observed in boys between ages 7 and 10 (average interaction effect: *β* = 0.035 [95% CI = 0.003–0.068]). Further, boys showed stronger associations between social–emotional unawareness and ADHD symptoms than girls between ages 7 and 9 (average interaction effect: *β* = 0.032 [95% CI = 0.003–0.061]), but this pattern reversed (i.e., girls showed stronger associations) at ages 11–12 (average interaction effect: *β* = −0.098 [95% CI = −0.194 to −0.002]). Girls also showed stronger associations between social–emotional unawareness and anxiety symptoms at ages 3–4 (average interaction effect: *β* = −0.023 [95% CI = −0.046 to −0.001]) and ages 11–12 (average interaction effect: *β* = −0.071 [95% CI = −0.127 to −0.010]). Further, the associations between repetitive mannerisms and anxiety symptoms were stronger in girls between ages 9 and 11 (average interaction effect: *β* = −0.048 [95% CI = −0.090 to −0.007]).

## 4 Discussion

The current study provides a novel and comprehensive investigation of the associations between autistic traits and co‐occurring EBCs in autistic children aged 2 to 12 years, addressing gaps in evidence related to measurement biases, longitudinal trends, and sex differences. These findings highlight the importance of considering finer‐grained behavioral domains and adopting developmental approaches to elucidate the intricate relations between autistic traits and EBCs. Below, we discuss each research aim.

### 4.1 Aim 1: Measurement Confounding Between Autistic Traits and EBCs

MNLFA revealed that the impact of child's sex and co‐occurring EBCs on parents' assessments of child's autistic traits was generally minor, except for a few items with varying thresholds. Our result of sex invariance aligns with previous research on autistic children using the SRS (Frazier et al. 2014; Uljarević et al. 2021). We further demonstrated that overall, the SRS measures autistic traits consistently across autistic children with varying levels of co‐occurring conditions. However, parents of children with higher anxiety levels tended to endorse certain items about social‐interaction difficulties, and items about repetitive mannerisms for children with more ADHD symptoms when controlling for overall level of autistic traits. Specifically, two items (“clings to adults, seems too dependent on them” and “wanders aimlessly from one activity to another”) demonstrated the most pronounced differential item functioning dependent on child's anxiety and ADHD symptoms, respectively. This indicates that these items may not effectively distinguish behavior indicating autism or anxiety/ADHD. For instance, clinging to adults in autistic children may be attributed to intolerance of uncertainty in the environment, which is potentially distinct from separation anxiety that can also be observed in non‐autistic children (Renno and Wood 2013). On the other hand, behavior such as wandering aimlessly observed in autistic children may not indicate inattentiveness as seen in ADHD, but rather reflect social inattention or unusual interest in sensory stimuli, suggesting a potentially distinct underlying mechanism (Hatch et al. 2023).

Compared to the overall minimal measurement bias related to sex and EBCs, the magnitude of age bias appeared to be larger. Some items were more likely to be endorsed by parents of younger children, while others were inclined towards those of older children. The different directions of item‐level bias may cancel out the impact of age bias on the latent construct or domain level (Teresi et al. 2012). Interestingly, the SRS domains most affected by age bias were, once again, social‐interaction difficulties and repetitive mannerisms. Further, the items identified with relatively large age DIF also encompassed the two above‐mentioned items flagged for bias related to EBCs. This suggests a need for more cautious interpretation of specific SRS domains and selected items when applying the measure to a diverse autistic population across developmental stages. It also highlights the advantage of further parsing the commonly used one‐domain or two‐domain (i.e., social‐communication difficulties and RRB) structure to finer‐grained domains, such as the current five‐factor structure (as modified from Frazier et al. 2014), to facilitate the identification and adjustment of measurement confounding.

Overall, our findings indicate that while the item‐level bias might not be influential at the construct level, caution is warranted when drawing inferences from specific items. Future iterations of parent‐report autism measures, like the SRS, which has been critiqued for its lack of specificity to autism (Charman et al. 2007; Hus et al. 2013), may consider refining or dropping items potentially conflated with co‐occurring EBCs and/or exhibiting age‐differential endorsement patterns. Developing adjusted scoring algorithms by weighing items that are less liable to measurement biases may also improve accuracy in assessing autistic traits.

### 4.2 Aim 2: Trajectories of Associations Between Autistic Traits and EBCs

#### 4.2.1 Autistic Traits and Anxiety/Affective Problems

Among the five domains of autistic traits, insistence on sameness showed the strongest overall associations with anxiety and affective problems, although this strong association appeared to diminish with age. The finding of the declining association aligns with the previous longitudinal evidence in the same sample on the association between insistence on sameness and anxiety symptoms (Baribeau et al. 2023), and may explain why such an association was not significant when examined with an age‐heterogeneous autistic sample in a prior study (Gotham et al. 2013). Further, a more noticeable decline in the association was observed between the domains of repetitive mannerisms and anxiety/affective problems. However, sex‐stratified trajectories revealed that autistic girls tended to demonstrate stable or intensifying patterns in the associations, with the sex difference becoming most pronounced during the transition into adolescence, especially for the associations with anxiety. This observation resonates with findings from a large autistic cohort, indicating that co‐occurring anxiety and/or ADHD was more strongly associated with RRBs in autistic adolescent females compared to their male and younger female counterparts (Wodka et al. 2022). Further, a recent mixed‐method study on autistic adolescents reported that autistic females were more likely to engage in stereotyped behavior when anxious, but not when excited (Cary et al. 2023). Thus, it is plausible that RRBs may serve a distinct role in adaptive self‐regulation to anxiety or other emotions for autistic females compared to males, particularly during late childhood and potentially extending through adolescence. Further research is merited to understand the differential associations between RRBs and internalizing problems in autistic females to inform more targeted support strategies.

In contrast to the RRB domains, the associations between social domains and anxiety/affective problems were generally stable, with peaks observed between ages 7 and 10 for the associations with affective problems. The timing of the peak is consistent with previous evidence on social‐communication difficulties and anxiety in a large sample including autistic and typically developing children (Pickard et al. 2017). Specifically, the associations with anxiety/affective problems appeared stronger for the domains of *social avoidance* and *social interaction difficulties*, which represent challenges arising from a lack of social skills, as compared to *social–emotional awareness*, which focuses more on social cognition and emotion recognition skills. This domain‐specific finding resonates with the prior evidence linking depression to core social‐communication difficulties but not to general social functioning in autistic youth (Pascoe et al. 2023). This highlights the necessity of distinguishing social–emotional (dys)functioning from social interaction difficulties and social avoidance under the broader social‐communication domain, with the latter two domains potentially being more contextual and linked to a lack of environmental support and reduced person‐environment fit (Chen et al. 2022; Lai et al. 2020).

Upon closer examination of the associations by sex, the strength of association between social‐communication domains and affective problems appeared to be stronger in autistic boys, while autistic girls exhibited increasing trends in the associations with anxiety symptoms. The peaking associations with affective problems during early school age in autistic boys align with the prior longitudinal evidence indicating that autistic boys tend to exhibit higher rates of depression than their female peers in early childhood (Gotham et al. 2015). This may be explained by the potentially moderating role of bullying or peer victimization at school, which was reported as a more robust predictor of depression in autistic boys than in girls (Greenlee et al. 2020). In autistic girls, the strengthened links between autistic traits and anxiety symptoms in late childhood might be attributed to coping strategies, such as masking, which are commonly reported in autistic females and associated with anxiety (Hull et al. 2021). These findings suggest a necessity for mental health support tailored to potential sex differences in experiencing and coping with environmental challenges, such as bullying and social stress, to address autistic people's unique needs and vulnerabilities when navigating various life stages.

#### 4.2.2 Autistic Traits and ADHD/Oppositional‐Defiant Problems

Compared to the generally decreasing pattern of associations between RRB and anxiety/affective problems, associations with ADHD/ODD problems were more stable, except for a reduced link between ADHD symptoms and social–emotional unawareness as well as repetitive mannerisms. This decreasing trend appears to contradict earlier findings of an increased link between autistic traits and ADHD in older children (Visser et al. 2016). However, this discrepancy may be attributed to the predominantly cross‐sectional designs and lack of consideration of subdomains within autistic traits in previous literature. The decreasing associations of ADHD with repetitive mannerisms and social–emotional unawareness observed in the current study may be explained by greater differentiation of ADHD symptoms and autistic traits in these domains among older children. This possibility is supported by a previous study, indicating that certain parent‐report items in these domains (e.g., offering comfort, complex body mannerisms) distinguished autistic children from those with ADHD during school age (Mouti et al. 2019). On the other hand, the associations with ODD symptoms were generally smaller but remained positive with all domains of autistic traits throughout childhood; however, the strength of associations was overall larger with the RRB domains, particularly the domain of insistence on sameness. Previous research with autistic children has also demonstrated links between RRBs, irritability, and aggression (Ibrahim et al. 2019; Kanne and Mazurek 2011), and those with higher insistence on sameness may be particularly at risk for ongoing oppositionality and externalizing behavior (Jasim and Perry 2023). Once again, these differential findings underscore the importance of scrutinizing finer‐grained domains when assessing the associations between autistic traits and co‐occurring EBCs.

Regarding sex differences, associations between social‐communication domains and ADHD/ODD symptoms appeared stronger in autistic boys, particularly around ages 7–9 during early school age. However, autistic boys showed more pronounced reductions in these associations upon entering late childhood, while the trend remained stable or increased in autistic girls—reflecting a pattern similar to those observed with anxiety problems. This pattern might be explained by the emergence of prototypical ADHD symptoms in autistic girls in late childhood or adolescence, which can be considered behavioral manifestations modulated by sex/gender‐related factors (Lai et al. 2022). While girls generally exhibit lower rates of conduct problems than boys, there may be a developmental progression in girls from ADHD symptoms to subsequent oppositional defiant disorder, which can be associated with self‐injurious behavior and the development of personality disorders or traits in preadolescent girls (Beauchaine et al. 2019; Lai et al. 2022). Further, our results suggested that autistic girls appeared to show more elevated associations between ADHD/ODD and RRB domains, especially insistence on sameness, than with social domains. Previous research has reported more elevated insistence on sameness and self‐injurious behavior in autistic girls (Antezana et al. 2019), as well as associations between externalizing behavior and ritualistic behavior, but not stereotypy (Jasim and Perry 2023). These suggest the relatively crucial role of insistence on sameness in both emotional (especially anxiety) and behavioral problems (Baribeau et al. 2023), which could be a key target for early identification, monitoring, and preventive interventions for mental health challenges, particularly among transition‐age autistic girls.

Overall, the associations between autistic traits and EBCs peaked in autistic boys around 7–9 years, coinciding with early school age, whereas for autistic girls, the peak occurred later around 10–12 years, potentially extending into adolescence. This sex‐differential pattern largely supports our hypotheses and highlights the importance of tailoring assessments and diagnostic procedures to specific developmental stages, while considering sex‐differential underlying mechanisms and adaptive strategies to better accommodate individual mental health needs across the autism spectrum and lifespan.

### 4.3 Limitations and Future Directions

While our study stands out as a novel longitudinal investigation into how associations between autistic traits and co‐occurring EBCs develop in autistic children, it is important to acknowledge several methodological limitations. First, our sample of autistic females in this study is relatively small (*n* = 62; 294 observations), potentially resulting in wider confidence intervals for the estimates and insufficient statistical power to detect sex effects at certain age points. Replications with larger samples, incorporating oversampling of autistic females, are essential for a robust evaluation of sex differences. Another limitation worth noting is our exclusion of intellectual functioning as a time‐varying covariate or an additional moderator. This limitation stems from constraints in the study design, including changing IQ measures and data collection schedules inconsistent with the primary measures (i.e., the SRS and CBCL). As intellectual functioning might affect the behavioral manifestation of autistic traits and emotional‐behavioral concerns (Mahony et al. 2023; McCauley et al. 2020), exploring the age‐varying effect of IQ in future research may offer a more thorough understanding of their intricate relations across behavioral domains. Last, our evaluation of autistic traits and EBCs depended on parent‐report measures, potentially inflating cross‐measure correlations. We also lacked information on whether our participants received clinician‐ascertained co‐occurring diagnoses, which limits our ability to verify parent‐reported symptoms. Despite our efforts to address measurement biases related to respondent characteristics, the reliance on parental reports should be taken into account when interpreting the current findings. Future research should consider replicating these findings with different cohorts using multi‐informant, multimodal measures for symptom quantification alongside diagnostic ascertainment.

## 5 Conclusion

The current investigation into the behavioral links between autism traits and EBCs revealed varying longitudinal patterns of associations by age, sex, and domain, indicating intricate dynamics throughout development in the manifestation of symptoms in autistic children. Early school age was marked by peak associations between autism traits and EBCs, highlighting the importance of screening for mental health needs and delivering related services during this period. Specifically, autistic boys generally showed stronger links between social‐communication difficulties and EBCs during early school age (ages 7–9), which tended to diminish upon entering late childhood (ages 10–12). In contrast, autistic girls exhibited overall stable or increasing association patterns, with more pronounced sex differences observed in the associations with anxiety symptoms in late childhood. This suggests that for autistic girls, the transition into adolescence represents a critical period for mental health support, particularly focusing on strategies to cope with anxiety linked to autistic traits. Our findings also highlight the advantages of identifying specific, less conflated constructs when measuring autistic traits, alongside employing a developmental approach to understand symptom coupling/decoupling at specific developmental stages. Gaining deeper insights in these areas will aid in optimizing supports tailored to the diverse emotional and behavioral needs of autistic people across the lifespan.

## Ethics Statement

The Pathways in ASD study was approved by the local research ethics boards at all recruitment sites.

## Conflicts of Interest

The authors declare no conflicts of interest.

## Supporting information


**Data S1.** Supporting Information.

**Data S2.** Supporting Information.

**Data S3.** Supporting Information.

## Data Availability

The data that support the findings of this study are available on request from the corresponding author. The data are not publicly available due to privacy or ethical restrictions.
