# Supplementary material for: Sex‐Differential Trajectories of Domain‐Specific Associations Between Autistic Traits and Co‐Occurring Emotional‐Behavioral Concerns in Autistic Children
Source: Autism Res. 2025 Mar 14;18(4):820–32. doi: 10.1002/aur.70018 (PMC12015799; doi:10.1002/aur.70018)
Supplement: Supplementary file 1 — Data S1. Supporting Information. [file AUR-18-820-s003.docx]

**Supplementary Tables and Figures**

**Table S1.** Descriptive statistics of primary measures by timepoint and sex

| **Time Points** | | **T1** | **T2** | **T3** | **T4** | **T5** | **T6** | **T7** | **T8** |
| --- | --- | --- | --- | --- | --- | --- | --- | --- | --- |
| Age Interval (months) | | 24-64 | 29-72 | 36-77 | 71-89 | 87-103 | 97-115 | 110-125 | 121-143 |
| Age Mean (SD) | M | 41.1 (9.4) | 48.0 (9.5) | 54.3 (9.2) | 79.1 (3.7) | 92.9 (2.7) | 104.7 (2.4) | 116.4 (2.4) | 129.2 (3.0) |
|  | F | 40.6 (9.5) | 48.0 (9.4) | 53.8 (9.4) | 79.8 (3.9) | 93.1 (2.8) | 104.8 (2.8) | 117.2 (3.1) | 128.8 (2.6) |
| N | M | 297 | 275 | 253 | 210 | 162 | 177 | 129 | 147 |
|  | F | 56 | 43 | 41 | 32 | 34 | 33 | 29 | 26 |
| Primary Measures - Standard Scores Mean (SD) | | | | | | | | | |
| SRS - Total | M | 74.5 (12.5) | 72.4 (13.2) | 72.0 (14.3) | 70.9 (15.3) | 72.4 (16.2) | 71.2 (16.2) | 72.4 (15.5) | 69.0 (14.8) |
|  | F | 84.1 (14.1) | 80.1 (15.2) | 79.3 (18.6) | 76.8 (14.4) | 81.3 (15.8) | 77.4 (18.0) | 82.2 (15.2) | 78.4 (18.8) |
| CBCL - Affective Problems | M | 58.4 (9.2) | 58.9 (9.1) | 57.9 (9.2) | 57.4 (8.6) | 57.6 (7.4) | 57.4 (7.4) | 58.5 (7.9) | 58.1 (7.7) |
|  | F | 59.4 (10.1) | 60.5 (9.7) | 59.5 (9.0) | 57.5 (10.0) | 57.8 (7.8) | 57.3 (8.6) | 59.5 (9.2) | 57.8 (8.4) |
| CBCL - Anxiety Problems | M | 56.7 (8.2) | 55.7 (8.2) | 55.4 (7.7) | 55.2 (7.4) | 57.8 (7.6) | 58.1 (8.3) | 59.0 (8.1) | 57.5 (7.9) |
|  | F | 59.7 (10.8) | 57.6 (8.6) | 57.8 (9.9) | 58.3 (11.0) | 60.1 (7.7) | 59.0 (9.7) | 61.2 (10.3) | 59.9 (8.5) |
| CBCL - Attention Deficit/Hyperactivity Problems | M | 57.7 (7.3) | 56.7 (6.7) | 55.8 (6.4) | 56.0 (7.2) | 59.3 (7.3) | 58.3 (7.8) | 59.1 (7.7) | 57.8 (7.8) |
|  | F | 58.3 (7.7) | 56.4 (6.8) | 57.0 (6.8) | 54.6 (5.7) | 61.3 (9.1) | 60.4 (9.2) | 61.8 (8.9) | 59.9 (8.1) |
| CBCL - Oppositional Defiant Problems | M | 56.2 (7.9) | 55.1 (7.0) | 54.7 (7.2) | 54.8 (7.5) | 55.9 (6.8) | 55.9 (7.7) | 56.0 (7.4) | 54.9 (6.6) |
|  | F | 56.7 (7.6) | 55.9 (7.0) | 55.4 (5.9) | 54.6 (7.6) | 59.4 (8.8) | 57.2 (8.1) | 58.4 (7.7) | 56.2 (6.8) |

**Figure S1.** Item/factor correspondence across different SRS factor structures

| Item # | 1-Factor | 2-Factor (DSM-5) | | 5-Factor A  (Treatment Scales) | | | | | 5-Factor B  (Frazier et al., 2014) | | | | | 5-Factor C  (Modified from Frazier et al., 2014) | | | | |
| --- | --- | --- | --- | --- | --- | --- | --- | --- | --- | --- | --- | --- | --- | --- | --- | --- | --- | --- |
|  |  | SCI | RRB | Awa | Cog | Comm | Mot | Mann | Avoid | ER | IR | IS | RM | Avoid | SEA | SID | IS | RM |
| 1 |  |  |  |  |  |  |  |  |  |  |  |  |  |  |  |  |  |  |
| 2 |  |  |  |  |  |  |  |  |  |  |  |  |  |  |  |  |  |  |
| 3 |  |  |  |  |  |  |  |  |  |  |  |  |  |  |  |  |  |  |
| 4 |  |  |  |  |  |  |  |  |  |  |  |  |  |  |  |  |  |  |
| 5 |  |  |  |  |  |  |  |  |  |  |  |  |  |  |  |  |  |  |
| 6 |  |  |  |  |  |  |  |  |  |  |  |  |  |  |  |  |  |  |
| 7 |  |  |  |  |  |  |  |  |  |  |  |  |  |  |  |  |  |  |
| 8 |  |  |  |  |  |  |  |  |  |  |  |  |  |  |  |  |  |  |
| 9 |  |  |  |  |  |  |  |  |  |  |  |  |  |  |  |  |  |  |
| 10 |  |  |  |  |  |  |  |  |  |  |  |  |  |  |  |  |  |  |
| 11 |  |  |  |  |  |  |  |  |  |  |  |  |  | Dropped | | | | |
| 12 |  |  |  |  |  |  |  |  |  |  |  |  |  |  |  |  |  |  |
| 13 |  |  |  |  |  |  |  |  |  |  |  |  |  |  |  |  |  |  |
| 14 |  |  |  |  |  |  |  |  |  |  |  |  |  |  |  |  |  |  |
| 15 |  |  |  |  |  |  |  |  |  |  |  |  |  |  |  |  |  |  |
| 16 |  |  |  |  |  |  |  |  |  |  |  |  |  |  |  |  |  |  |
| 17 |  |  |  |  |  |  |  |  |  |  |  |  |  |  |  |  |  |  |
| 18 |  |  |  |  |  |  |  |  |  |  |  |  |  |  |  |  |  |  |
| 19 |  |  |  |  |  |  |  |  |  |  |  |  |  |  |  |  |  |  |
| 20 |  |  |  |  |  |  |  |  |  |  |  |  |  |  |  |  |  |  |
| 21 |  |  |  |  |  |  |  |  |  |  |  |  |  |  |  |  |  |  |
| 22 |  |  |  |  |  |  |  |  |  |  |  |  |  |  |  |  |  |  |
| 23 |  |  |  |  |  |  |  |  |  |  |  |  |  |  |  |  |  |  |
| 24 |  |  |  |  |  |  |  |  |  |  |  |  |  |  |  |  |  |  |
| 25 |  |  |  |  |  |  |  |  |  |  |  |  |  |  |  |  |  |  |
| 26 |  |  |  |  |  |  |  |  |  |  |  |  |  |  |  |  |  |  |
| 27 |  |  |  |  |  |  |  |  |  |  |  |  |  |  |  |  |  |  |
| 28 |  |  |  |  |  |  |  |  |  |  |  |  |  |  |  |  |  |  |
| 29 |  |  |  |  |  |  |  |  |  |  |  |  |  |  |  |  |  |  |
| 30 |  |  |  |  |  |  |  |  |  |  |  |  |  |  |  |  |  |  |
| 31 |  |  |  |  |  |  |  |  |  |  |  |  |  |  |  |  |  |  |
| 32 |  |  |  |  |  |  |  |  |  |  |  |  |  |  |  |  |  |  |
| 33 |  |  |  |  |  |  |  |  |  |  |  |  |  |  |  |  |  |  |
| 34 |  |  |  |  |  |  |  |  |  |  |  |  |  |  |  |  |  |  |
| 35 |  |  |  |  |  |  |  |  |  |  |  |  |  |  |  |  |  |  |
| 36 |  |  |  |  |  |  |  |  |  |  |  |  |  |  |  |  |  |  |
| 37 |  |  |  |  |  |  |  |  |  |  |  |  |  |  |  |  |  |  |
| 38 |  |  |  |  |  |  |  |  |  |  |  |  |  |  |  |  |  |  |
| 39 |  |  |  |  |  |  |  |  |  |  |  |  |  |  |  |  |  |  |
| 40 |  |  |  |  |  |  |  |  |  |  |  |  |  |  |  |  |  |  |
| 41 |  |  |  |  |  |  |  |  |  |  |  |  |  |  |  |  |  |  |
| 42 |  |  |  |  |  |  |  |  |  |  |  |  |  |  |  |  |  |  |
| 43 |  |  |  |  |  |  |  |  |  |  |  |  |  | Dropped | | | | |
| 44 |  |  |  |  |  |  |  |  |  |  |  |  |  |  |  |  |  |  |
| 45 |  |  |  |  |  |  |  |  |  |  |  |  |  |  |  |  |  |  |
| 46 |  |  |  |  |  |  |  |  |  |  |  |  |  |  |  |  |  |  |
| 47 |  |  |  |  |  |  |  |  |  |  |  |  |  |  |  |  |  |  |
| 48 |  |  |  |  |  |  |  |  |  |  |  |  |  |  |  |  |  |  |
| 49 |  |  |  |  |  |  |  |  |  |  |  |  |  |  |  |  |  |  |
| 50 |  |  |  |  |  |  |  |  |  |  |  |  |  |  |  |  |  |  |
| 51 |  |  |  |  |  |  |  |  |  |  |  |  |  |  |  |  |  |  |
| 52 |  |  |  |  |  |  |  |  |  |  |  |  |  |  |  |  |  |  |
| 53 |  |  |  |  |  |  |  |  |  |  |  |  |  |  |  |  |  |  |
| 54 |  |  |  |  |  |  |  |  |  |  |  |  |  |  |  |  |  |  |
| 55 |  |  |  |  |  |  |  |  |  |  |  |  |  |  |  |  |  |  |
| 56 |  |  |  |  |  |  |  |  |  |  |  |  |  |  |  |  |  |  |
| 57 |  |  |  |  |  |  |  |  |  |  |  |  |  |  |  |  |  |  |
| 58 |  |  |  |  |  |  |  |  |  |  |  |  |  |  |  |  |  |  |
| 59 |  |  |  |  |  |  |  |  |  |  |  |  |  |  |  |  |  |  |
| 60 |  |  |  |  |  |  |  |  |  |  |  |  |  |  |  |  |  |  |
| 61 |  |  |  |  |  |  |  |  |  |  |  |  |  |  |  |  |  |  |
| 62 |  |  |  |  |  |  |  |  |  |  |  |  |  |  |  |  |  |  |
| 63 |  |  |  |  |  |  |  |  |  |  |  |  |  |  |  |  |  |  |
| 64 |  |  |  |  |  |  |  |  |  |  |  |  |  |  |  |  |  |  |
| 65 |  |  |  |  |  |  |  |  |  |  |  |  |  |  |  |  |  |  |

Note: SCI=Social Communication/Interaction, RRB=Restricted/Repetitive Behavior, Awa=Social Awareness, Cog=Social Cognition, Comm=Social Communication, Mot=Social Motivation, Mann=Autistic Mannerisms, Avoid=Social Avoidance, ER=Emotion Recognition, IR=Interpersonal Relatedness, IS=Insistence on Sameness, RM=Repetitive Mannerisms, SEA=Social-Emotional Awareness, SID= Social-Interaction Difficulties.

**Table S2.** SRS five-factor model**:** Standardized factor loadings and internal consistency (63 items^*^)

|  | | Sample 1 | Sample 2 | Sample 3 |
| --- | --- | --- | --- | --- |
| N | | 388 | 279 | 249 |
| Age in years Mean (SD) | | 3.45 (.79) | 6.70 (.44) | 9.81 (.67) |
| Item | | Standardized Factor Loadings | | |
| **Factor 1: Social Avoidance (9 items)** | | | | |
| 6 | Would rather be alone than with others | .539 | .677 | .638 |
| 16 | Avoids eye contact, or has unusual eye contact | .610 | .455 | .563 |
| 18 | Child does not attempt to interact with other children | .629 | .701 | .753 |
| 23 | Does not join group activities unless told to do so | .595 | .734 | .784 |
| 27 | Avoids starting social interactions with peers or adults | .683 | .720 | .734 |
| 34 | Avoids people who want to be emotionally close to him/her | .601 | .707 | .615 |
| 36 | Has difficulty “relating” to adults | .677 | .773 | .695 |
| 37 | Has difficulty “relating” to peers | .753 | .915 | .855 |
| 60 | Is emotionally distant, doesn’t show his/her emotions | .609 | .683 | .644 |
| Composite Reliability (ω) | | .858 | .902 | .897 |
| **Factor 2: Social-Emotional Awareness (15 items)** | | | | |
| 3 | Seems self-confident when interacting with others | .449 | .458 | .601 |
| 7 | Is aware of what others are thinking or feeling | .596 | .675 | .676 |
| 12 | Is able to communicate his or her feelings to others in words or gestures | .563 | .752 | .766 |
| 15 | Is able to understand the meaning of other people’s tone of voice and facial expressions | .581 | .687 | .497 |
| 17 | Recognizes when something is unfair | .509 | .700 | .751 |
| 21 | Is able to imitate others’ actions | .461 | .454 | .472 |
| 22 | Plays appropriately with children his/her age | .736 | .845 | .895 |
| 26 | Offers comfort to others when they are sad | .591 | .647 | .641 |
| 32 | Wants to be changed when diaper or underwear are soiled or wet | .396 | .613 | .567 |
| 38 | Responds appropriately to mood changes in others | .581 | .656 | .777 |
| 40 | Is imaginative, good at pretending losing touch with reality | .640 | .718 | .604 |
| 45 | Focuses attention to where others are looking or listening | .697 | .667 | .613 |
| 48 | Has a sense of humor, understands jokes | .571 | .716 | .745 |
| 52 | Knows when he/she is talking too loud or making too much noise | .606 | .586 | .603 |
| 55 | Knows when is too close to someone | .551 | .547 | .657 |
|  | Composite Reliability (ω) | .879 | .917 | .921 |
| **Factor 3:** **Social-Interaction Difficulties (12 items)** | | | | |
| 2 | Expressions on his/her face don’t match what he/she is saying | .476 | .655 | .572 |
| 5 | Doesn’t recognize when others are trying to take advantage of him/her | .514 | .607 | .760 |
| 9 | Clings to adults, seems too dependent on them | .322 | .469 | .547 |
| 10 | Unable to pick up on any of the meaning of conversations | .486 | .451 | .628 |
| 13 | Is slow or awkward in turn-taking interactions with peers | .623 | .618 | .644 |
| 14 | Is not well coordinated in physical activities | .319 | .449 | .277 |
| 19 | Gets frustrated trying to get ideas across in conversations | .555 | .569 | .605 |
| 33 | Is socially awkward | .790 | .714 | .752 |
| 35 | Has trouble keeping up with the flow of a normal interaction | .639 | .833 | .842 |
| 44 | Doesn’t understand how events are related to one another | .494 | .744 | .793 |
| 51 | Responds to clear, direct questions in ways that don’t seem to make sense | .423 | .513 | .570 |
| 57 | Other children do not like to play with him/her | .589 | .410 | .485 |
| Composite Reliability (ω) | | .819 | .866 | .888 |
| **Factor 4: Insistence on Sameness (15 items)** | | | | |
| 1 | Seems much more fidgety in social situations than when alone | .619 | .574 | .635 |
| 4 | When under stress, child seems to go on “auto-pilot” | .701 | .740 | .795 |
| 24 | Has more difficulty with changes in routine | .729 | .786 | .760 |
| 28 | Thinks or talks about the same thing over and over | .636 | .416 | .639 |
| 30 | Becomes upset in a situation with lots of things going on | .734 | .774 | .810 |
| 31 | Can’t get his/her mind off something once he/she starts thinking about it | .712 | .632 | .759 |
| 42 | Seems overly sensitive to sounds, textures, or smells | .607 | .549 | .597 |
| 46 | Has overly serious facial expressions | .557 | .557 | .550 |
| 49 | Does extremely well at a few tasks, but does not do as well at others | .636 | .805 | .647 |
| 53 | Talks to people with an unusual tone of voice (like a robot) | .586 | .485 | .493 |
| 58 | Concentrates too much on parts of things | .515 | .479 | .578 |
| 59 | Is overly suspicious | .356 | .457 | .474 |
| 61 | Is inflexible, has a hard time changing his/her mind | .673 | .723 | .734 |
| 62 | Gives unusual or illogical reasons for doing things | .641 | .425 | .534 |
| 64 | Is too tense in social settings | .683 | .647 | .706 |
| Composite Reliability (ω) | | .907 | .898 | .917 |
| **Factor 5:** **Repetitive Mannerisms (12 items)** | | | | |
| 8 | Behaves in ways which seem strange or bizarre | .743 | .831 | .863 |
| 20 | Has strange way of playing with toys | .616 | .716 | .760 |
| 25 | Doesn’t seem to mind being “out of step” | .456 | .522 | .500 |
| 29 | Is regarded by other children as odd or weird | .740 | .801 | .803 |
| 39 | Has a restricted (or unusually narrow) range of interests | .712 | .722 | .748 |
| 41 | Wanders aimlessly from one activity to another | .621 | .647 | .628 |
| 47 | Is too silly or laughs inappropriately | .548 | .670 | .610 |
| 50 | Has repetitive, odd behaviors such as hand flapping or rocking | .521 | .673 | .703 |
| 54 | Seems to react to people as if they are objects | .634 | .750 | .724 |
| 56 | Walks in between two people who are talking | .504 | .545 | .644 |
| 63 | Touches others in an unusual way | .566 | .681 | .618 |
| 65 | Stares or gazes off into space | .603 | .604 | .614 |
| Composite Reliability (ω) | | .875 | .913 | .915 |

*Note. Two items were dropped: Item 43 (separates easily from caregivers) was dropped due to its weak factor loading across models. Item 11 (has good self-confidence) was dropped due to its high residual covariance with item 3 (seems self-confident when interacting with others).

**Table S3.** Inter-factor correlations (Pearson’s *r*) of SRS factors

|  | F1 | F2 | F3 | F4 | F5 |
| --- | --- | --- | --- | --- | --- |
| F1: Social Avoidance | - |  |  |  |  |
| F2: Social-Emotional (Un)Awareness^*^ | .61 | - |  |  |  |
| F3: Social-Interaction Difficulties | .72 | .46 | - |  |  |
| F4: Insistence on Sameness | .62 | .29 | .73 | - |  |
| F5: Repetitive Mannerisms | .41 | .56 | .73 | .72 | - |

Note. ^*^Item responses were reversely coded.

**Table S4.** Item-level results of moderated nonlinear factor analysis: Associations (Pearson’s *r*) between covariates and structural parameters for autistic traits averaged across calibration samples

| # | Item | Age | | Age^2^ | | Male | | Affective | | Anxiety | | ADHD | | ODD | |
| --- | --- | --- | --- | --- | --- | --- | --- | --- | --- | --- | --- | --- | --- | --- | --- |
|  |  | Int | Load | Int | Load | Int | Load | Int | Load | Int | Load | Int | Load | Int | Load |
| **SRS Factor 1: Social Avoidance (9 items)** | | | | | | | | | | | | | | | |
| 6 | Would rather be alone than with others | 0.037 | 0.035 | 0.036 | 0.057 | 0.029 | 0.049 | 0.048 | 0.025 | 0.019 | 0.031 | 0.082 | 0.021 | 0.041 | 0.021 |
| 16 | Avoids eye contact, or has unusual eye contact | 0.029 | 0.031 | 0.059 | 0.037 | 0.035 | 0.063 | 0.038 | 0.028 | 0.067 | 0.043 | 0.065 | 0.030 | 0.047 | 0.029 |
| 18 | Child does not attempt to interact with other children | **0.110** | 0.025 | 0.027 | 0.032 | 0.053 | 0.020 | 0.060 | 0.034 | 0.078 | 0.018 | 0.047 | 0.040 | 0.061 | 0.038 |
| 23 | Does not join group activities unless told to do so | 0.089 | 0.070 | 0.034 | 0.022 | 0.040 | 0.033 | 0.034 | 0.029 | 0.019 | 0.044 | 0.038 | 0.057 | 0.058 | 0.021 |
| 27 | Avoids starting social interactions with peers or adults | **0.154** | 0.060 | 0.045 | 0.046 | 0.029 | 0.071 | 0.065 | 0.088 | 0.023 | 0.039 | 0.055 | 0.049 | 0.027 | 0.034 |
| 34 | Avoids people who want to be emotionally close to him/her | 0.063 | 0.047 | 0.035 | 0.029 | 0.044 | 0.021 | **0.105** | 0.027 | 0.054 | 0.030 | 0.054 | 0.071 | 0.071 | 0.069 |
| 36 | Has difficulty “relating” to adults | 0.064 | 0.015 | 0.038 | 0.068 | 0.045 | 0.031 | 0.052 | 0.046 | **0.103** | 0.031 | 0.010 | 0.055 | 0.015 | 0.032 |
| 37 | Has difficulty “relating” to peers | 0.050 | 0.030 | 0.035 | 0.066 | 0.068 | 0.074 | 0.034 | 0.042 | 0.022 | 0.031 | 0.038 | 0.070 | 0.012 | 0.034 |
| 60 | Is emotionally distant, doesn’t show his/her emotions | 0.083 | 0.037 | 0.042 | 0.036 | 0.069 | 0.035 | 0.054 | 0.040 | 0.041 | 0.064 | 0.029 | 0.014 | 0.087 | 0.032 |
| **SRS Factor 2: Social-Emotional (Un)Awareness (15 items)** | | | | | | | | | | | | | |  |  |
| # | Item | Age | | Age^2^ | | Male | | Affective | | Anxiety | | ADHD | | ODD | |
|  |  | Int | Load | Int | Load | Int | Load | Int | Load | Int | Load | Int | Load | Int | Load |
| 3 | Seems self-confident when interacting with others | 0.085 | 0.079 | 0.054 | 0.028 | 0.037 | 0.022 | 0.032 | 0.028 | **0.173** | 0.022 | **0.150** | 0.055 | 0.026 | 0.038 |
| 7 | Is aware of what others are thinking or feeling | 0.017 | 0.024 | 0.057 | 0.045 | 0.057 | 0.024 | 0.027 | 0.049 | 0.093 | 0.078 | 0.061 | 0.058 | 0.049 | 0.075 |
| 12 | Is able to communicate his or her feelings to others in words or gestures | 0.044 | 0.036 | 0.059 | 0.039 | 0.043 | 0.005 | 0.086 | 0.022 | 0.024 | 0.061 | 0.055 | 0.030 | 0.029 | 0.012 |
| 15 | Is able to understand the meaning of other people’s tone of voice and facial expressions | **0.137** | 0.035 | 0.062 | 0.060 | 0.048 | 0.038 | 0.055 | 0.013 | 0.063 | 0.090 | 0.081 | 0.036 | 0.068 | 0.060 |
| 17 | Recognizes when something is unfair | **0.113** | 0.048 | 0.021 | 0.033 | 0.050 | 0.073 | 0.052 | 0.040 | **0.113** | 0.036 | 0.068 | 0.042 | 0.047 | 0.043 |
| 21 | Is able to imitate others’ actions | 0.066 | 0.042 | **0.105** | 0.058 | 0.027 | 0.063 | 0.034 | 0.033 | 0.019 | 0.068 | 0.032 | 0.025 | 0.057 | 0.037 |
| 22 | Plays appropriately with children his/her age | 0.028 | 0.038 | 0.040 | 0.040 | 0.024 | 0.013 | 0.087 | 0.036 | 0.040 | 0.039 | 0.061 | 0.025 | 0.087 | 0.026 |
| 26 | Offers comfort to others when they are sad | 0.089 | 0.025 | 0.082 | 0.054 | 0.042 | 0.020 | 0.083 | 0.005 | 0.032 | 0.021 | 0.067 | 0.047 | 0.032 | 0.015 |
| 32 | Wants to be changed when diaper or underwear are soiled or wet | **0.129** | 0.036 | 0.081 | 0.063 | 0.038 | 0.035 | 0.070 | 0.042 | 0.049 | 0.065 | 0.036 | 0.029 | 0.084 | 0.038 |
| 38 | Responds appropriately to mood changes in others | 0.081 | 0.021 | 0.079 | 0.063 | 0.024 | 0.038 | 0.053 | 0.036 | 0.048 | 0.067 | 0.040 | 0.021 | 0.086 | 0.054 |
| 40 | Is imaginative, good at pretending losing touch with reality | 0.089 | 0.034 | **0.129** | 0.021 | **0.167** | 0.025 | 0.028 | 0.050 | 0.017 | 0.035 | 0.041 | 0.054 | 0.059 | 0.006 |
| 45 | Focuses attention to where others are looking or listening | 0.032 | 0.049 | 0.049 | 0.040 | 0.013 | 0.039 | 0.017 | 0.041 | 0.061 | 0.035 | 0.056 | 0.034 | 0.079 | 0.005 |
| 48 | Has a sense of humor, understands jokes | 0.048 | 0.059 | 0.026 | 0.024 | 0.057 | 0.044 | 0.025 | 0.053 | 0.024 | 0.050 | 0.031 | 0.022 | 0.026 | 0.023 |
| 52 | Knows when he/she is talking too loud or making too much noise | 0.041 | 0.029 | 0.043 | 0.029 | 0.027 | 0.028 | 0.023 | 0.075 | 0.030 | 0.015 | **0.103** | 0.019 | 0.022 | 0.009 |
| 55 | Knows when is too close to someone | 0.070 | 0.025 | 0.055 | 0.033 | 0.024 | 0.024 | 0.012 | 0.038 | 0.043 | 0.055 | 0.077 | 0.050 | 0.055 | 0.028 |
| **SRS Factor 3: Social-Interaction Difficulties (12 items)** | | | | | | | | | | | | | | | |
| # | Item | Age | | Age^2^ | | Male | | Affective | | Anxiety | | ADHD | | ODD | |
|  |  | Int | Load | Int | Load | Int | Load | Int | Load | Int | Load | Int | Load | Int | Load |
| 2 | Expressions on his/her face don’t match what he/she is saying | **0.131** | 0.097 | 0.052 | 0.049 | 0.011 | 0.020 | 0.086 | 0.047 | 0.030 | **0.127** | 0.032 | 0.055 | 0.073 | 0.034 |
| 5 | Doesn’t recognize when others are trying to take advantage of him/her | **0.191** | 0.026 | **0.109** | 0.016 | 0.028 | 0.042 | 0.040 | 0.036 | **0.120** | 0.069 | 0.045 | **0.117** | 0.055 | 0.086 |
| 9 | Clings to adults, seems too dependent on them | **0.212** | 0.033 | **0.107** | 0.076 | 0.054 | 0.043 | 0.010 | 0.040 | **0.282** | 0.022 | 0.047 | 0.051 | 0.031 | 0.031 |
| 10 | Unable to pick up on any of the meaning of conversations | 0.098 | 0.057 | 0.049 | 0.023 | 0.033 | 0.023 | 0.099 | 0.042 | 0.049 | 0.045 | 0.055 | 0.029 | 0.042 | 0.031 |
| 13 | Is slow or awkward in turn-taking interactions with peers | **0.183** | 0.033 | **0.112** | 0.042 | 0.034 | 0.010 | 0.031 | 0.066 | 0.017 | 0.059 | 0.091 | 0.030 | 0.020 | 0.037 |
| 14 | Is not well coordinated in physical activities | 0.045 | 0.037 | 0.038 | 0.010 | 0.060 | 0.014 | 0.032 | 0.032 | 0.070 | 0.011 | 0.055 | 0.033 | 0.031 | 0.028 |
| 19 | Gets frustrated trying to get ideas across in conversations | 0.091 | 0.029 | 0.080 | 0.047 | 0.076 | 0.057 | 0.021 | 0.078 | 0.066 | 0.022 | 0.088 | 0.050 | **0.130** | 0.087 |
| 33 | Is socially awkward | **0.113** | 0.007 | 0.078 | 0.059 | 0.040 | 0.016 | 0.042 | 0.027 | 0.025 | 0.064 | 0.063 | 0.046 | 0.038 | 0.039 |
| 35 | Has trouble keeping up with the flow of a normal interaction | **0.127** | 0.040 | 0.017 | 0.023 | 0.057 | 0.028 | 0.020 | 0.028 | 0.083 | 0.045 | 0.081 | 0.047 | 0.055 | 0.013 |
| 44 | Doesn’t understand how events are related to one another | **0.135** | 0.028 | 0.040 | 0.063 | 0.029 | 0.020 | 0.031 | 0.034 | 0.084 | 0.042 | 0.036 | 0.086 | 0.031 | 0.055 |
| 51 | Responds to clear, direct questions in ways that don’t seem to make sense | 0.059 | 0.015 | 0.064 | 0.044 | 0.020 | 0.024 | 0.092 | 0.078 | 0.032 | 0.070 | 0.047 | 0.018 | 0.009 | 0.061 |
| 57 | Other children do not like to play with him/her | **0.292** | 0.050 | **0.113** | 0.045 | 0.040 | 0.028 | 0.034 | 0.020 | 0.057 | 0.028 | 0.032 | 0.026 | 0.088 | 0.022 |
| **SRS Factor 4: Insistence on Sameness (15 items)** | | | | | | | | | | | | | | | |
| # | Item | Age | | Age^2^ | | Male | | Affective | | Anxiety | | ADHD | | ODD | |
|  |  | Int | Load | Int | Load | Int | Load | Int | Load | Int | Load | Int | Load | Int | Load |
| 1 | Seems much more fidgety in social situations than when alone | 0.042 | 0.016 | 0.053 | 0.032 | 0.007 | 0.030 | 0.035 | 0.030 | 0.065 | 0.041 | 0.050 | 0.053 | 0.050 | 0.029 |
| 4 | When under stress, child seems to go on “auto-pilot” | 0.039 | 0.073 | 0.024 | 0.014 | 0.020 | 0.034 | 0.013 | 0.028 | 0.046 | 0.040 | 0.035 | 0.043 | 0.029 | 0.042 |
| 24 | Has more difficulty with changes in routine | **0.132** | 0.052 | 0.033 | 0.066 | 0.047 | 0.015 | 0.026 | 0.058 | 0.011 | 0.026 | 0.016 | 0.043 | 0.055 | 0.049 |
| 28 | Thinks or talks about the same thing over and over | **0.161** | 0.043 | 0.078 | 0.024 | 0.071 | 0.092 | 0.095 | 0.041 | 0.004 | 0.030 | 0.061 | 0.025 | 0.044 | 0.034 |
| 30 | Becomes upset in a situation with lots of things going on | 0.069 | 0.049 | 0.056 | 0.052 | 0.017 | 0.070 | 0.045 | 0.098 | 0.021 | 0.079 | 0.061 | 0.047 | 0.024 | 0.024 |
| 31 | Can’t get his/her mind off something once he/she starts thinking about it | 0.047 | 0.063 | 0.052 | 0.037 | 0.023 | 0.024 | 0.059 | 0.027 | 0.014 | 0.041 | 0.027 | 0.028 | 0.058 | 0.023 |
| 42 | Seems overly sensitive to sounds, textures, or smells | 0.047 | 0.075 | 0.065 | 0.067 | 0.036 | 0.046 | 0.109 | 0.045 | 0.056 | 0.052 | 0.036 | 0.062 | 0.085 | 0.036 |
| 46 | Has overly serious facial expressions | 0.025 | 0.023 | 0.047 | 0.046 | 0.046 | 0.030 | **0.113** | 0.009 | 0.042 | 0.039 | 0.013 | 0.050 | 0.041 | 0.028 |
| 49 | Does extremely well at a few tasks, but does not do as well at others | 0.045 | 0.044 | 0.021 | 0.084 | 0.033 | 0.028 | 0.058 | 0.033 | 0.077 | 0.025 | **0.144** | 0.017 | 0.073 | 0.042 |
| 53 | Talks to people with an unusual tone of voice (like a robot) | **0.167** | 0.068 | 0.092 | 0.074 | 0.085 | 0.057 | 0.039 | 0.034 | 0.040 | 0.039 | 0.041 | 0.048 | 0.036 | 0.054 |
| 58 | Concentrates too much on parts of things | 0.030 | 0.046 | 0.020 | 0.055 | 0.035 | 0.026 | 0.078 | 0.047 | 0.011 | 0.037 | 0.026 | 0.035 | 0.042 | 0.008 |
| 59 | Is overly suspicious | 0.094 | 0.008 | 0.044 | 0.021 | 0.036 | 0.029 | 0.053 | 0.030 | 0.046 | 0.027 | 0.060 | 0.074 | 0.020 | 0.054 |
| 61 | Is inflexible, has a hard time changing his/her mind | 0.097 | 0.030 | 0.041 | 0.055 | 0.061 | 0.022 | 0.025 | 0.028 | 0.027 | 0.028 | 0.033 | 0.055 | **0.138** | 0.050 |
| 62 | Gives unusual or illogical reasons for doing things | **0.128** | 0.034 | 0.079 | 0.020 | 0.046 | 0.015 | 0.055 | 0.018 | 0.070 | 0.060 | 0.065 | 0.018 | 0.017 | 0.013 |
| 64 | Is too tense in social settings | 0.083 | 0.100 | 0.051 | 0.088 | 0.038 | 0.072 | 0.023 | 0.020 | 0.096 | 0.028 | 0.052 | 0.039 | 0.048 | 0.022 |
| **SRS Factor 5: Repetitive Mannerisms (12 items)** | | | | | | | | | | | | | | | |
| # | Item | Age | | Age^2^ | | Male | | Affective | | Anxiety | | ADHD | | ODD | |
|  |  | Int | Load | Int | Load | Int | Load | Int | Load | Int | Load | Int | Load | Int | Load |
| 8 | Behaves in ways which seem strange or bizarre | 0.028 | 0.052 | 0.019 | 0.047 | 0.037 | 0.073 | 0.048 | 0.018 | 0.054 | 0.054 | 0.071 | 0.067 | 0.065 | 0.071 |
| 20 | Has strange way of playing with toys | **0.180** | 0.051 | 0.038 | 0.035 | 0.077 | 0.030 | 0.033 | 0.014 | 0.009 | 0.067 | 0.027 | 0.014 | 0.020 | 0.028 |
| 25 | Doesn’t seem to mind being “out of step” | 0.033 | 0.051 | 0.029 | 0.032 | 0.042 | 0.065 | 0.052 | 0.024 | 0.029 | 0.027 | 0.041 | 0.046 | 0.038 | 0.044 |
| 29 | Is regarded by other children as odd or weird | **0.299** | 0.057 | 0.080 | 0.059 | 0.022 | 0.035 | 0.024 | 0.065 | 0.030 | 0.050 | 0.028 | 0.064 | 0.036 | 0.074 |
| 39 | Has a restricted (or unusually narrow) range of interests | 0.066 | 0.054 | 0.048 | 0.032 | **0.134** | 0.038 | **0.101** | 0.038 | 0.031 | 0.008 | 0.079 | 0.049 | 0.051 | 0.048 |
| 41 | Wanders aimlessly from one activity to another | **0.202** | 0.085 | 0.028 | 0.032 | 0.024 | 0.043 | 0.017 | 0.047 | 0.077 | 0.057 | **0.249** | 0.043 | 0.059 | 0.051 |
| 47 | Is too silly or laughs inappropriately | **0.269** | 0.019 | 0.080 | 0.015 | 0.046 | 0.035 | 0.045 | 0.088 | 0.019 | 0.027 | 0.096 | 0.025 | 0.032 | 0.052 |
| 50 | Has repetitive, odd behaviors such as hand flapping or rocking | 0.091 | 0.050 | **0.102** | 0.029 | 0.084 | 0.050 | 0.059 | 0.036 | 0.060 | 0.014 | 0.090 | 0.036 | 0.032 | 0.053 |
| 54 | Seems to react to people as if they are objects | 0.075 | 0.023 | 0.034 | 0.039 | 0.014 | 0.011 | 0.050 | 0.059 | 0.068 | 0.024 | 0.022 | 0.027 | 0.040 | 0.057 |
| 56 | Walks in between two people who are talking | 0.038 | 0.034 | 0.048 | 0.079 | 0.037 | 0.074 | 0.067 | 0.049 | 0.019 | 0.041 | **0.133** | 0.031 | 0.015 | 0.032 |
| 63 | Touches others in an unusual way | 0.022 | 0.024 | 0.078 | 0.035 | 0.060 | 0.008 | 0.034 | 0.044 | 0.032 | 0.068 | 0.066 | 0.018 | 0.024 | 0.056 |
| 65 | Stares or gazes off into space | 0.038 | 0.067 | 0.046 | 0.045 | 0.032 | 0.074 | 0.066 | 0.055 | 0.032 | 0.046 | 0.072 | 0.034 | 0.006 | 0.029 |

Note. INT=intercept bias; Load=factor loading bias


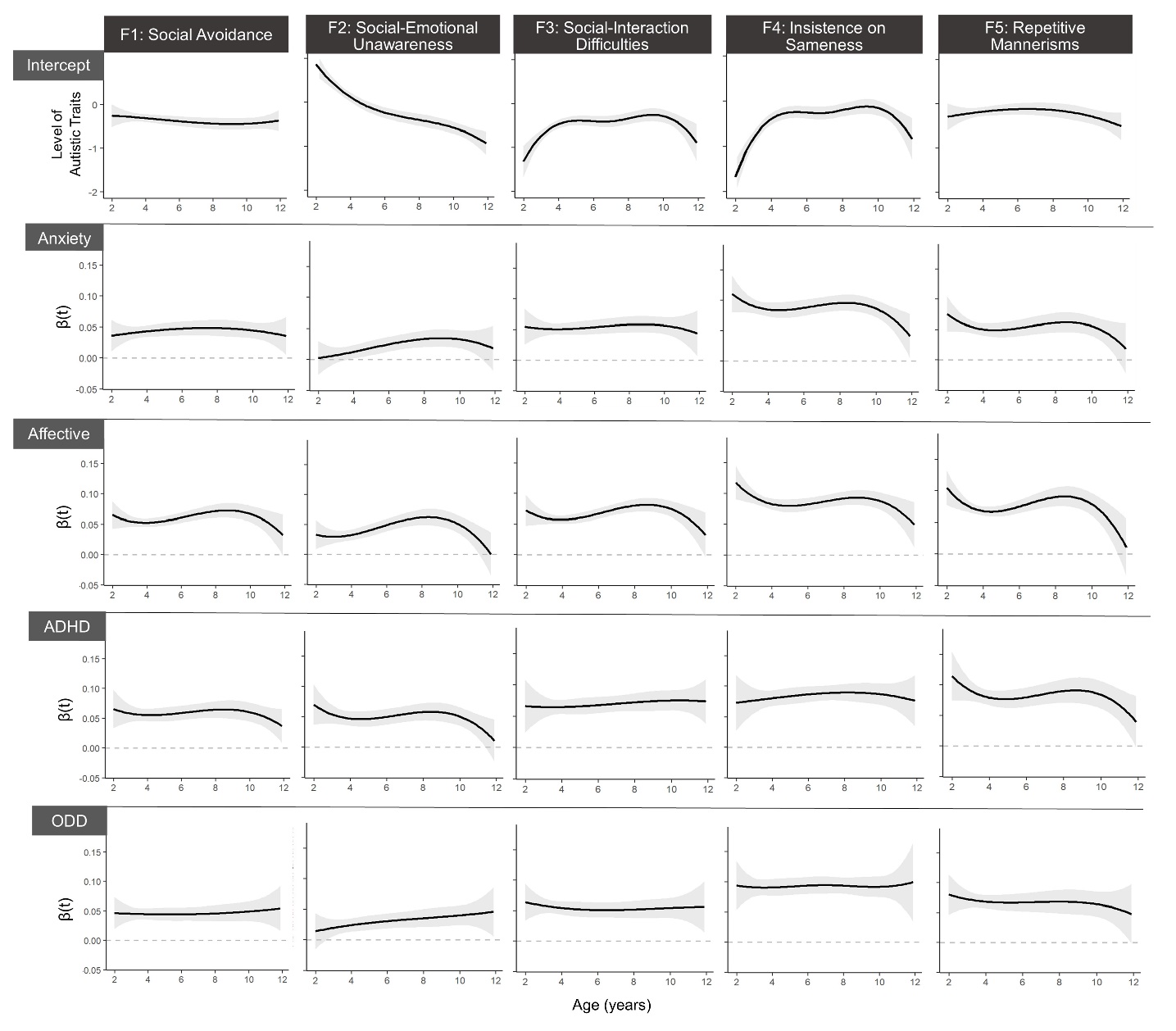
**Figure S2.** Time-varying main effect of CBCL domains (row) on SRS factors (column) in the full sample without sex stratification

Note. The first row presents results from the intercept-only models of nonparametric regression of autistic traits (SRS factors) on age, which can be interpreted as the mean trajectories of autistic traits. Rows two through five present the age-varying main effects of each CBCL subscale on each SRS factor, which were modeled as spline regression coefficients and confidence intervals. Significance is indicated when the 95% confidence intervals do not overlap with zero.

**Table S5.** Summary of the age-varying associations between SRS and CBCL domains

|  | All (N=389; 1944 observations) | | | | | Boys (N=327; 1650 observations) | | | | | Girls (N=62; 294 observations) | | | | |
| --- | --- | --- | --- | --- | --- | --- | --- | --- | --- | --- | --- | --- | --- | --- | --- |
|  | Average Time-varying Effects (β) | | | Age Points of Max/Min Estimates (years) | | Average Time-varying Effects (β) | | | Age Points of Max/Min Estimates (years) | | Average Time-varying Effects (β) | | | Age Points of Max/Min Estimates (years) | |
|  | Ages 2-5 | Ages  6-8 | Ages  9-12 | Max | Min | Ages 2-5 | Ages  6-8 | Ages  9-12 | Max | Min | Ages  2-5 | Ages  6-8 | Ages  9-12 | Max | Min |
| SRS F1: Social Avoidance | | | | | | | | | | | | | | | |
| *Anxiety* | .043 | .048 | .042 | 7.4 | 11.9 | .044 | .050 | .041 | 7.0 | 11.9 | .036 | .043 | .047 | 10.9 | 2.0 |
| *Affective* | .056 | .069 | .058 | 8.7 | 11.9 | .057 | .073 | .061 | 8.7 | 11.9 | .051 | .050 | .048 | 2.0 | 11.2 |
| *ADHD* | .058 | .063 | .054 | 2.0 | 11.9 | .060 | .069 | .058 | 8.7 | 11.9 | .049 | .041 | .037 | 4.3 | 9.3 |
| *ODD* | .045 | .046 | .051 | 11.9 | 5.3 | .045 | .049 | .050 | 2.0 | 3.4 | .053 | .030 | .043 | 2.0 | 6.6 |
| SRS F2: Social-Emotional Unawareness | | | | | | | | | | | | | | | |
| *Anxiety* | .013 | .031 | .029 | 8.9 | 2.0 | .010 | .030 | .022 | 8.7 | 11.9 | .025 | .033 | .052 | 11.2 | 2.0 |
| *Affective* | .036 | .059 | .038 | 8.4 | 11.9 | .030 | .068 | .049 | 11.9 | 2.0 | .040 | .039 | .036 | 10.0 | 11.2 |
| *ADHD* | .052 | .056 | .040 | 2.0 | 11.9 | .053 | .061 | .037 | 2.0 | 11.9 | .044 | .033 | .049 | 11.2 | 8.0 |
| *ODD* | .024 | .034 | .042 | 11.9 | 2.0 | .024 | .038 | .039 | 9.4 | 2.0 | .023 | .018 | .043 | 11.2 | 7.0 |
| SRS F3: Social-Interaction Difficulties | | | | | | | | | | | | | | | |
| *Anxiety* | .052 | .057 | .053 | 8.7 | 11.9 | .058 | .060 | .051 | 2.0 | 11.9 | .031 | .046 | .059 | 11.2 | 2.0 |
| *Affective* | .062 | .077 | .064 | 8.7 | 11.9 | .062 | .080 | .065 | 8.6 | 11.9 | .057 | .063 | .062 | 2.0 | 11.2 |
| *ADHD* | .066 | .071 | .075 | 10.4 | 3.5 | .069 | .074 | .073 | 9.0 | 3.3 | .049 | .058 | .081 | 11.2 | 4.4 |
| *ODD* | .056 | .052 | .055 | 2.0 | 6.5 | .055 | .056 | .052 | 2.0 | 11.9 | .062 | .033 | .056 | 11.2 | 8.0 |
| SRS F4: Insistence on Sameness | | | | | | | | | | | | | | | |
| *Anxiety* | .090 | .093 | .075 | 2.0 | 11.9 | .094 | ,093 | .068 | 2.0 | 11.9 | .075 | .096 | .107 | 9.5 | 4.3 |
| *Affective* | .088 | .090 | .076 | 2.0 | 11.9 | .089 | .088 | .075 | 2.0 | 11.9 | .085 | .094 | .091 | 2.0 | 11.2 |
| *ADHD* | .080 | .089 | .084 | 8.1 | 2.0 | .078 | .083 | .071 | 9.7 | 11.9 | .078 | .104 | .112 | 11.2 | 2.0 |
| *ODD* | .096 | .091 | .082 | 2.0 | 11.9 | .094 | .092 | .082 | 2.0 | 11.9 | .094 | .096 | .101 | 11.2 | 2.0 |
| SRS F5: Repetitive Mannerisms | | | | | | | | | | | | | | | |
| *Anxiety* | .055 | .059 | .046 | 2.0 | 11.9 | .058 | .058 | .037 | 2.0 | 11.9 | .050 | .069 | .090 | 10.1 | 4.5 |
| *Affective* | .076 | .086 | .061 | 2.0 | 11.9 | .077 | .087 | .061 | 2.0 | 11.9 | .068 | .080 | .075 | 2.0 | 11.2 |
| *ADHD* | .085 | .087 | .072 | 2.0 | 11.9 | .086 | .087 | .069 | 2.0 | 11.9 | .084 | .097 | .094 | 2.0 | 4.0 |
| *ODD* | .070 | .068 | .060 | 2.0 | 11.9 | .070 | .071 | .061 | 2.0 | 11.9 | .072 | .060 | .062 | 2.0 | 11.2 |
